# Development and Kinematic Verification of a Finite Element Model for the Lumbar Spine: Application to Disc Degeneration

**DOI:** 10.1155/2013/705185

**Published:** 2012-12-05

**Authors:** Elena Ibarz, Antonio Herrera, Yolanda Más, Javier Rodríguez-Vela, José Cegoñino, Sergio Puértolas, Luis Gracia

**Affiliations:** ^1^Department of Mechanical Engineering, University of Zaragoza, 50018 Zaragoza, Spain; ^2^Department of Surgery, University of Zaragoza, 50009 Zaragoza, Spain; ^3^Department of Orthopaedic Surgery and Traumatology, Miguel Servet University Hospital, 50009 Zaragoza, Spain; ^4^Aragón Health Sciences Institute, 50009 Zaragoza, Spain; ^5^Engineering and Architecture School, University of Zaragoza, María de Luna 3, 50018 Zaragoza, Spain

## Abstract

The knowledge of the lumbar spine biomechanics is essential for clinical applications. Due to the difficulties to experiment on living people and the irregular results published, simulation based on finite elements (FE) has been developed, making it possible to adequately reproduce the biomechanics of the lumbar spine. A 3D FE model of the complete lumbar spine (vertebrae, discs, and ligaments) has been developed. To verify the model, radiological images (X-rays) were taken over a group of 25 healthy, male individuals with average age of 27.4 and average weight of 78.6 kg with the corresponding informed consent. A maximum angle of 34.40° is achieved in flexion and of 35.58° in extension with a flexion-extension angle of 69.98°. The radiological measurements were 33.94 ± 4.91°, 38.73 ± 4.29°, and 72.67°, respectively. In lateral bending, the maximum angles were 19.33° and 23.40 ± 2.39, respectively. In rotation a maximum angle of 9.96° was obtained. The model incorporates a precise geometrical characterization of several elements (vertebrae, discs, and ligaments), respecting anatomical features and being capable of reproducing a wide range of physiological movements. Application to disc degeneration (L5-S1) allows predicting the affection in the mobility of the different lumbar segments, by means of parametric studies for different ranges of degeneration.

## 1. Introduction

Lumbar pain currently represents a serious problem due to its socioeconomic impact and repercussions. Degenerative disc disease is the most common cause of lumbar pain [1]. Factors which can have an influence on the degenerative process are, amongst others, the loads supported [2] (which in addition can activate the enzymatic processes which play a part in the degeneration [3]), the movements in flexion [4], and the genetics of each individual [5, 6]. 

The lumbar spine is a complex structure in biomechanical terms. It has to combine flexibility to allow three-dimensional movements and stability to protect the nervous structures, whilst maintaining a biplanar equilibrium in the erect posture with minimum muscular effort. On the other hand, the spine is a viscoelastic structure which modifies its mechanical properties in relation to the intensity of the load [7]. There are numerous studies to determine the ranges of lumbar spine mobility, in addition to others that analyse the forces and loads that influence the movements and displacements produced. The biomechanics of the lumbar spine have been studied both on corpses [8–11] and “in vivo” using simple or biplanar radiographs [12–14] or other associated methods [15]. Other studies to determine mobility have used a variety of systems, associating studies with TV and computer [16], CT [17], electrogoniometer [18], video fluoroscope [19, 20], NMR [21–23], the inclinometer [24], or measurements with goniometer and the distraction between anatomical structures [25]. Animal spines have also been used [26] for laboratory tests, although there are notable differences between these and human spines [27]. The complexity of the lumbar spine, along with the variability of each individual, conditions the difficulty and reproducibility of “in vivo” or “in vitro” biomechanical studies. 

Due to the difficulties of being able to experiment on living people, the irregular results that have been published and the differences between human and animal spines, simulation models have been developed through the use of finite elements (FE). These models make it possible to study the lumbar spine in both physiological and pathological conditions, whenever the model is precise enough to adequately reproduce the biomechanics of the lumbar spine. This method, in existence since 1956 [28], was popularized among the scientific community during the 60s decade [29] and has proved to be adequate for the study of the functionality of a physiological unit as complex as the lumbar spine. There are numerous studies dedicated to simulating the different behavioural aspects of the lumbar spine, from a global biomechanical level to the more specific performance problems of some elements or even different pathologies [30–40]. The majority of the models concerning specific problems consider a unique functional unit or two functional units at the most [30, 38–40]. The availability of a model of the complete lumbar spine would allow a complete nonlinear biomechanical analysis of a healthy lumbar spine, as a step towards studying the consequences of disc degeneration and the effects produced by the implantation of different lumbar fixations or disc prosthesis, not only in a specific functional unit but in any level along the lumbar spine, even multiple degeneration levels and fixations.

Concerning disc degeneration, different types of studies have been reported. So, in [41] an in vitro study is presented for 44 corpse specimens, classifying degeneration levels according Thompson criteria [42]. A complete revision of the main factors affecting disc degeneration from a clinical point of view is presented in [43]. A discussion about reliability of in vitro and in vivo models for the study of disc degeneration is included in [44]. In the field of simulation, a model of poroelastic materials, both for the nucleus pulposus and annulus fibrosus, considering only a functional unit is presented in [45]. Other authors apply different mechanical models for the behaviour of the degenerated disc, but including only a functional unit in most of the cases [35, 40, 46–49].

The objective of this work was to develop and verify a complete three-dimensional FE model of the lumbar spine from L1 to Sacrum with the corresponding intervertebral discs, as well as all the ligaments which intervene in the biomechanical behaviour, reproduced with the greater anatomical detail. By means of this model, lumbar spine standard movements can be simulated, verifying the model with the results obtained in radiographic measurements carried out on healthy individuals and comparing it with published results. After kinematic verification, the model has been applied to the study of disc degeneration obtaining the difference of mobility between healthy and pathologic conditions.

## 2. Materials and Methods

**Figure 1:**
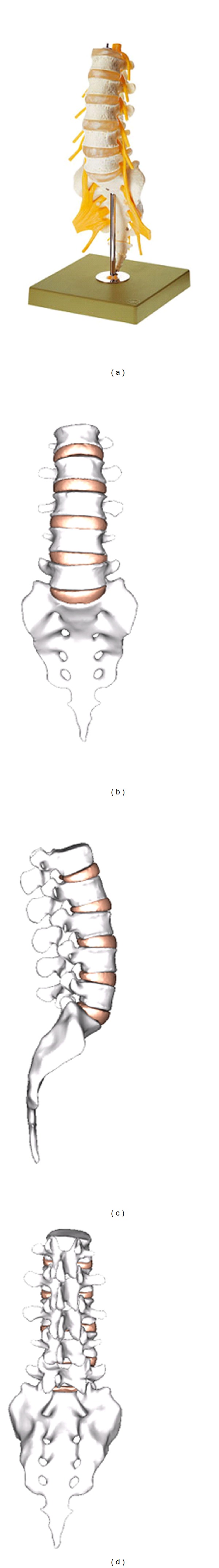
Anatomic model of the lumbar spine and complete geometric model (frontal, lateral, and dorsal views).

In order to get a model as accurate as possible of the lumbosacral spine, a mixed technique has been used. The starting point for obtaining a precise outer geometric representation of the discs and vertebrae is an anatomical model, trademark Somso QS-15 (Figure 1). The individual parts of this model are scanned using a Roland PICZA laser scanner and processed using the programs Dr. Picza 3 and 3D Editor. Each one of these parts is then positioned to achieve the complete model, in accordance to the spatial placement obtained by means of a 3D CT scan in healthy individuals. Figure 1 shows the geometrical accuracy obtained by means of that procedure, with the modelled geometry reproducing all the anatomical relevant aspects. Then the transition from the zone of exterior cortical bone to the zone of interior cancellous bone was obtained by means of statistical averages from CTs of vertebrae in healthy individuals, with results similar to those mentioned in the bibliography [50]. This method combines high accuracy in the external form with an excellent definition of internal interfaces and a perfect correlation among the different anatomical structures.

The mesh of the vertebrae is made by means of tetrahedral elements with quadratic approximation in the I-deas program [51] with a size thin enough to allow a smooth transition from the zone of exterior cortical bone to the zone of interior cancellous bone. The mesh of the discs is essential for the correct reproduction of the biomechanical behaviour of the lumbar spine; in order to do this, each disc is divided into nucleus pulposus and annulus fibrosus with commonly accepted dimensions [50]. Each part is meshed separately, the nucleus by means of tetrahedra and the annulus by means of hexahedra and prisms with quadratic approximation. The mesh sizes must concord with each other and with the vertebrae. Later, nine layers (outer and four double crossed) of concentric fibres are added to the annulus. These layers are modelled by means of tension-only elements, included in the hexahedra matrix, with variable orientation from the most internal to the most external (Figure 3), ranging from 35° to 80°, respecting at most the anatomical disposition [50, 52]. 

Finally, the ligaments (anterior longitudinal, posterior longitudinal, interspinous, flavum, supraspinous, intertransverse, and iliolumbar) are modelled by means of tetrahedra and prisms with quadratic approximation; in addition, membrane elements have been used for capsular ligaments. The dimensions of those soft tissues correspond to average anatomical measurements [50, 52] (Figure 3). The number of finite elements for every part is shown in Table 1. The total number of elements of the final mesh, obtained after a sensitivity analysis, is 196553. To this respect a mesh refinement was performed in order to achieve a convergence towards a minimum of the potential energy, both for the whole model and for each of its components, with a tolerance of 1% between consecutive meshes.

The bone and ligament properties were taken from the bibliography. Concerning the bone, in [30] it is demonstrated that the centre of the vertebrae is less rigid than in the exterior zone. For this reason the vertebrae are divided into four areas with variable modulus of elasticity (Figure 4). In addition, the corresponding properties are used for the cancellous bone (Table 1).

In the discs, the nucleus pulposus behaves like a noncompressible fluid, which upon being compressed expands towards the exterior tractioning the fibers of the annulus. This behaviour was simulated by means of the hyperelastic Mooney-Rivlin model (incompressible) incorporated in the Abaqus materials library [53]. The fibres of the annulus exhibit a nonlinear only tension behaviour approximated using different linear models for each layer considering their respective range of deformation [30]. The materials of the matrix and cartilage of the apophyses were simulated as elastic materials. Finally, the different ligaments present nonlinear only tension behaviour, included as a bilinear model in the strain range (Table 1) as with most of the reported FEM studies [30, 38]. 

Four basic movements will be studied: flexion, extension, lateral bending, and rotation (Figure 5), from which any movement of the spine can be obtained. As boundary conditions displacements in the wings of sacrum have been prevented. In all cases the starting point is a compression of 400 *n*, which simulates the precompression due to the body weight. That compression was applied as a follower load from L1 to L5 as is done in [54]. This a standard option in the Abaqus software [53]. Later, by means of an iterative procedure based on optimization techniques, the forces and moments on each vertebra were adjusted until the degrees of rotation in every vertebral segment were achieved according to the specific movement, taking in to account three fundamental muscular groups for flexion-extension [55]: psoas major as local muscle and rectum and erector spinae as global muscles. For lateral bending and rotation, oblique and multifidus muscles were added. The procedure calculates the force at every considered muscle along the paths of their respective movements (Figure 6, for flexion). Then the associated energy is evaluated as

(1) $$\begin{array}{c} W_{Fi}=\oint_{C_{i}}\vec{F_{i}}\cdot d\vec{s_{i}} \end{array}$$

for the forces (local muscles) and

(2) $$\begin{array}{c} W_{Mj}=\int_{0}^{\alpha_{i}}{\vec{M}}_{j}\cdot d\vec{\theta } \end{array}$$

for the moments (global muscles). The total energy is minimized for every movement:

(3) $$\begin{array}{c} \min W=\sum_{i=1}^{N}W_{Fj}+\sum_{j=1}^{M}W_{Mj} \end{array}$$

with *N* and *M* the number of local and global muscles, respectively, considered in the analyzed movement. As a restriction for the minimization problem, all the forces and moments must be nonnegative.

The correct interaction between the different elements (vertebrae, discs, and ligaments) is essential. For inserting the ligaments, the guidelines set in the anatomy manuals have been followed. Conditions of union between the different vertebral bodies and the corresponding intervertebral discs have been established, as it is the most representative of the real anatomy. Because vertebrae and discs were meshed in a separate and independent way in order to get a more accurate definition of the different regions in each of them, the common surfaces between the vertebrae and the discs demand an adequate surface congruency to avoid stress concentrations in isolated points. Then, a joint condition must be established (TIE option, standard in Abaqus software [53]). Finally, contact conditions have been established between the different apophyses which provide a global stability, taking into account that an important part of the loads are transmitted through them. Capsular ligaments were also included in order to a better simulation of physiological conditions. The calculation and postprocessing were carried out using the Abaqus program.

For every movement, the changes in the relative position of the vertebrae in respect to the sacrum are measured by means of perpendicular lines on the upper face of each vertebra, associated with four knots on which the monitoring is carried out (Figure 7). In the same way, another two reference lines are defined on the sacrum. So, for every vertebra, the reference coordinates are [56]:

(1) *frontal plane*  (initial node (4); final node (5)):
  (4) $$\begin{array}{c} N_{f1}^{Li}\equiv N_{f1}\left(\begin{array}{ccc} X_{f1} & Y_{f1} & Z_{f1} \end{array}\right), \end{array}$$
  (5) $$\begin{array}{c} N_{f2}^{Li}\equiv N_{f2}\left(\begin{array}{ccc} X_{f2} & Y_{f2} & Z_{f2} \end{array}\right). \end{array}$$
(2) *sagittal plane* (initial node (6); final node (7)):
  (6) $$\begin{array}{c} N_{s1}^{Li}\equiv N_{s1}\left(\begin{array}{ccc} X_{s1} & Y_{s1} & Z_{s1} \end{array}\right), \end{array}$$
  (7) $$\begin{array}{c} N_{s2}^{Li}\equiv N_{s2}\left(\begin{array}{ccc} X_{s2} & Y_{s2} & Z_{s2} \end{array}\right). \end{array}$$
  Using the formulae of analytical geometry, the properties of both lines can be obtained. For the length,
(3) *frontal plane:*
  (8) $$\begin{array}{c} L_{f}=\sqrt{{\left(X_{f2}-X_{f1}\right)}^{2}+{\left(Y_{f2}-Y_{f1}\right)}^{2}+{\left(Z_{f2}-Z_{f1}\right)}^{2}}, \end{array}$$
(4) *sagittal plane: *
  (9) $$\begin{array}{c} L_{s}=\sqrt{{\left(X_{s2}-X_{s1}\right)}^{2}+{\left(Y_{s2}-Y_{s1}\right)}^{2}+{\left(Z_{s2}-Z_{s1}\right)}^{2}}, \end{array}$$
  and for the directional cosines, in the general case,
(5) *frontal plane:*
  (10) $$\begin{array}{c} l_{f}=\frac{X_{f2}-X_{f1}}{L_{f}},\;\;m_{f}=\frac{Y_{f2}-Y_{f1}}{L_{f}},\;\;\;\;\;\\ n_{f}=\frac{Z_{f2}-Z_{f1}}{L_{f}}, \end{array}$$
(6) *sagittal plane: *
  (11) $$\begin{array}{c} l_{s}=\frac{X_{s2}-X_{s1}}{L_{s}},\;\;m_{s}=\frac{Y_{s2}-Y_{s1}}{L_{s}},\;\;n_{s}=\frac{Z_{s2}-Z_{s1}}{L_{s}}. \end{array}$$

The same applies for the sacrum. Then the relative angle with respect to the sacrum can be obtained by means of the scalar product, applying the above equations to every particular case as the following:

(1) flexo-extension (sagittal plane, *YZ*):
  (12) $$\begin{array}{c} \cos\alpha_{\text{FE}}\left(Li-S\right)=m_{s}^{Li}m_{s}^{S}+n_{s}^{Li}n_{s}^{S}, \end{array}$$
(2) lateral bending (frontal plane, *XZ*):
  (13) $$\begin{array}{c} \cos\alpha_{\text{LB}}\left(Li-S\right)=l_{f}^{Li}l_{f}^{S}+n_{f}^{Li}n_{f}^{S}, \end{array}$$
(3) axial rotation (horizontal plane, *XY*):
  (14) $$\begin{array}{c} \cos\alpha_{\text{AR}}\left(Li-S\right)=l_{f}^{Li}l_{f}^{S}+m_{f}^{Li}m_{f}^{S}. \end{array}$$

Computing the above values for every vertebra and sacrum, it is possible to determine their relative positions in space, both in the undeformed and deformed configurations, corresponding to the different analyzed movements.

In order to collect the radiological measurements which make it possible to contrast and validate the developed model, a group of 25 healthy volunteers, male individuals with an average age of 27.4 years, ranging from 23 to 33, and an average weight of 78.6 Kg, ranging from 72.1 to 81.7, with the corresponding informed consent were taken. Two radiographic techniques have been used: (a) standing, starting from a neutral position and performing movements of flexion, extension, and lateral bending; (b) the radiographs of flexion and extension were repeated placing the individuals in sitting position with hips bent at 90° above the torso and knees also bent at 90°, with a dense, rubber, and foam device at the level of the abdomen. No significant differences between the values of flexion and extension were found with respect to those obtained in the standing position.

For the measurements on the radiological images, we proceed at a graphic level with the same methodology of comparing the relative positions of common points. Lines are depicted at the top of every vertebra, and then the final position after movement is compared with the equivalent line in the standing position for the different movements (Figure 8). The radiological monitoring of the torsion has not been performed due to the fact that reliable measurements cannot be obtained from frontal, dorsal, and/or lateral images as those used in the rest of movements. The in vivo study was used to verify the accuracy obtained for the movement of individual vertebrae. In fact, there is a lot of sets of values for muscle force that produce the global movement, but only one of them is coherent with all the individual movements.

In the case of disc degeneration, MRI can detect disc space narrowing, osteophyte formation, vacuum phenomena, and water content. The incompressibility is reduced due to nucleus dehydration, and the disc deformation implies some compressibility. From a mechanical point of view, two effects have to be taken into account: a loss of disc rise and a loss of tension in the ligaments, basically in the anterior and in the posterior ones. The degenerative process induces a certain degree of instability in the affected unit depending on the degree of degeneration. From the healthy model, pathological conditions were simulated in the L5-S1 disc diminishing the nucleus compressibility and modifying the stiffness in the different elements according Table 2. In this case, a normalized moment of 15 m·N has been used for every movement except for axial rotation (6 m·N) acting on L1, according to the range used in the specialized literature. The objective is not to realize a sophisticated model for disc behaviour, as is done in specialized studies involving just a functional unit [35, 46, 47], but to analyze the influence of disc degeneration in the global mobility of the lumbar spine.

## 3. Results and Discussion

The results concerning radiological measurements are included in Table 3. The results obtained from the simulation model and from radiological measurements are depicted in Figure 9 for the four movements analysed. It can be seen that in every case a progressive movement of the vertebrae is produced as the distance to the sacrum increases, so that the global movements are increasing in the order L5 → L4 → L3 → L2 → L1. Concerning the radiological movements, the range of values obtained coincides with the results of the simulation by means of FE, as well as with the physiological values [50, 52].

Revising every movement, the evolution of the values obtained for flexion can be seen in Figure 9(a), compared to the radiological measurements and physiological values of reference. A maximum angle of 34.40° is achieved (L1), and an accurate correspondence can be observed with the radiological measurements (33.94 ± 4.91°) as well as a good approximation to the physiological values [50, 52].

The evolution of the values obtained for extension can be seen in Figure 9(b), comparing them again with the radiological measurements and physiological values of reference. A maximum angle of 35.58° is achieved (L1), and a very good agreement with the radiological measurements (38.73 ± 4.29°) as well as a good approximation to the physiological values can be observed. The results for the complete movement of flexion-extension are shown in Figure 9(c), with a whole flexion-extension angle of 69.98° (mean value of 72.67° in the radiological measurements). Logically a good degree of approximation is maintained with both the results of the radiological measurement and physiological values [50, 52], both in the global movement and in the ones corresponding to every vertebra.

In Figure 9(d) the values obtained for lateral bending and its evolution are shown. Once more the values are compared with the radiological measurements and with the physiological values of [50, 52] and show a very high degree of approximation again. A maximum angle of 19.33° was reached (23.40 ± 2.39° in the radiological measurements).

Finally, the values obtained for the movement of torsion are shown in Figure 9(e). In this case the values obtained by means of simulation are compared with the physiological values of [50, 52] and once again show a good concurrence. A maximum angle of 9.96° was reached in this movement.

Finally, concerning recent references, in Mosnier [57] are collected a lot of results, corresponding to different in vivo tests. In Figure 10 can be seen a comparison between those results and the values obtained in the present work. A good agreement is obtained for the different movements.

As for the tensional state, due to the anatomical accuracy of the model, precise stress distributions can be obtained for either part. So, as an example, Figure 11 shows the distribution of von Mises stress (MPa) in vertebrae and sacrum in the movement of torsion, as it relates to a movement which has been studied less than the rest of movements in the specialized literature. A progressive increase in the tensional level in the order L5 → L4 → L3 → L2 → L1 is observed. The distribution of maximum shear tension (MPa) in disc L5-S1 in extension where the effect of shear is more marked is also shown. Some localized zones of maximum shear in the posterior zone of the annulus fibrosus are detected, with a noticeable tensional discontinuity between the annulus and the nucleus, as corresponds to materials with very different rigidities. All the obtained values are according to the previously published ranges [18, 54, 58].

For the discs, in Figure 12 the stresses (tension) on the different fibers of the annulus fibrosus are shown. The blue-coloured fibers are situated in the zones of compression, hence they are not working. In the movement of flexion, the maximum tensions in the posterior fibers are reached, whilst in extension the maximum tensions correspond to the anterior fibers. In lateral bending the fibers on the opposite side to the inclination of the torso are loaded. Finally, in the movement of torsion, the fibers tensioned along the five discs form a helix, which corresponds to the optimum mechanism of resistance to torsion of any mechanical element. In the same way, the precise stress distribution for every component in the model (vertebrae, nucleus, annulus, and ligaments) can be obtained for every analyzed movement or even different combinations of the basic movements. 

In the simulated pathologic conditions, a higher mobility is detected at every vertebral level when comparing with healthy conditions, according to [59]. So in Figure 13 a comparison of the deformed configurations for the different movements is presented, and in Figure 14 the numerical values for the rotation at different vertebral levels are included. Finally, in Figure 15 a comparative diagram shows the mobility differences between healthy and pathological conditions, with values of 19.4% for flexion, 23.3% for extension, 29.1% for lateral bending, and 10.3% for axial rotation.

Despite one can find in the literature previous validated models of the lumbar spine with a good agreement with experimental tests [54], the developed model incorporates improvements in some aspects. So a precise geometrical characterization (without simplifications) of all of the constituent elements (vertebrae, discs, ligaments, and cartilages) according to anatomical features is done. This allows a better characterization of the ligaments-apophyses interaction, avoiding the local effects produced by one-dimensional elements in the 3D models.

The model also provides a suitable definition concerning conditions of interaction between elements (vertebrae-discs interaction, vertebrae/discs-ligaments interaction, and contact between articular apophyses). This gives rise to a nonlinear behaviour of the whole model, with results that reliably reproduce those obtained in other studies. There are models in the literature much better in the properties characterization (porous materials, hypoelasticity, incompressible fluid, etc.), but such models concern to only one element (vertebra, disc) or a functional unit (two vertebrae and intervertebral disc) at most [30, 38–40].

**Figure 2:**
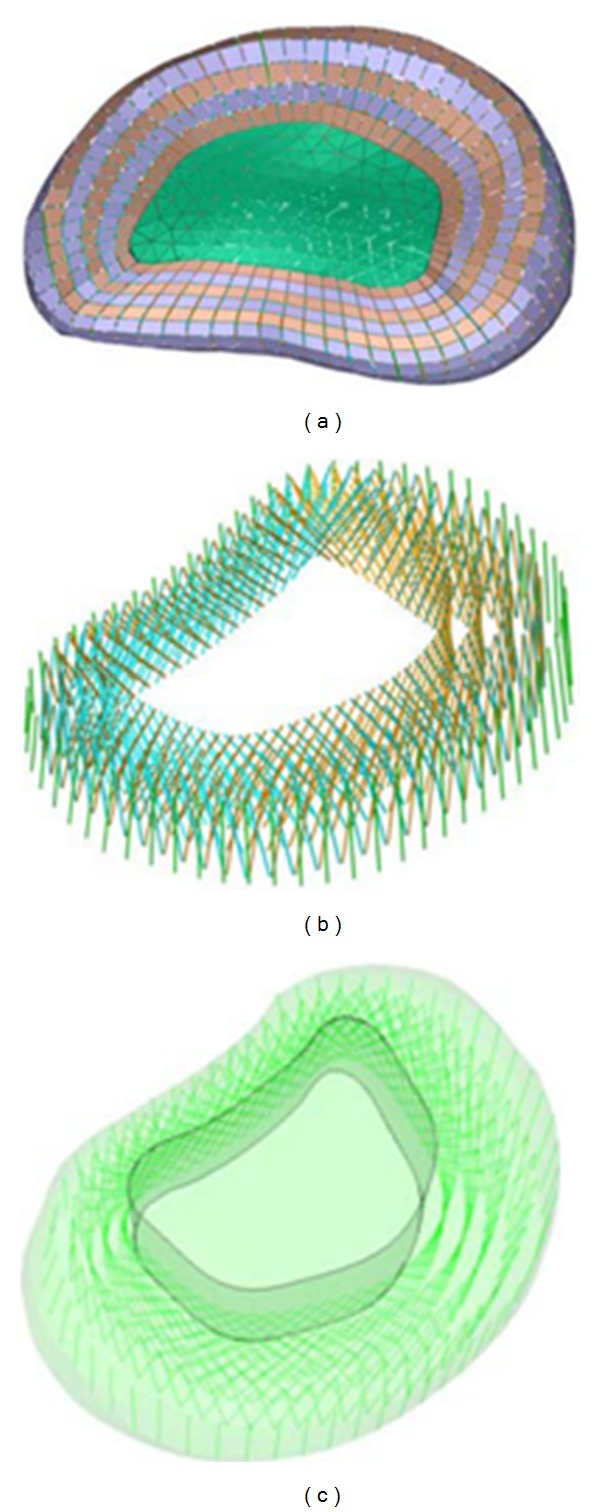
Complete FE model including vertebrae, discs, and ligaments (frontal, lateral, and dorsal views).

**Figure 3:**
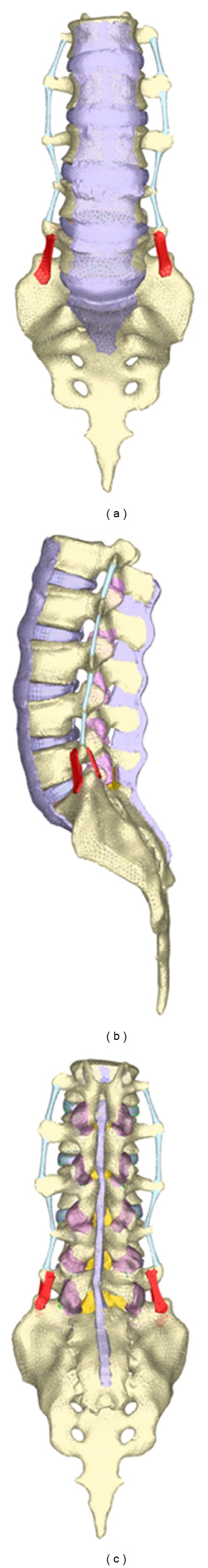
Model of the intervertebral disk and its layers of fibres.

**Figure 4:**
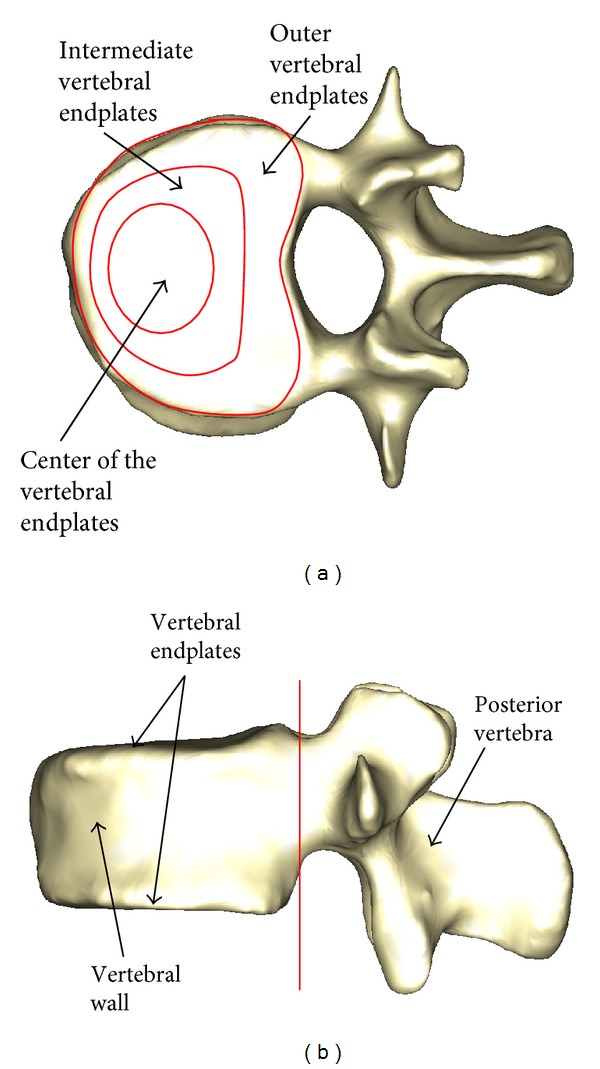
Zones of different elastic properties in the vertebral body.

**Figure 5:**
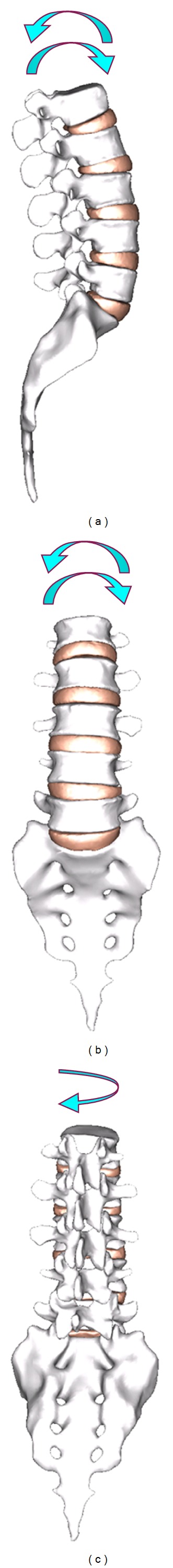
Simulated movements: flexion-extension, lateral bending, and axial rotation.

**Figure 6:**
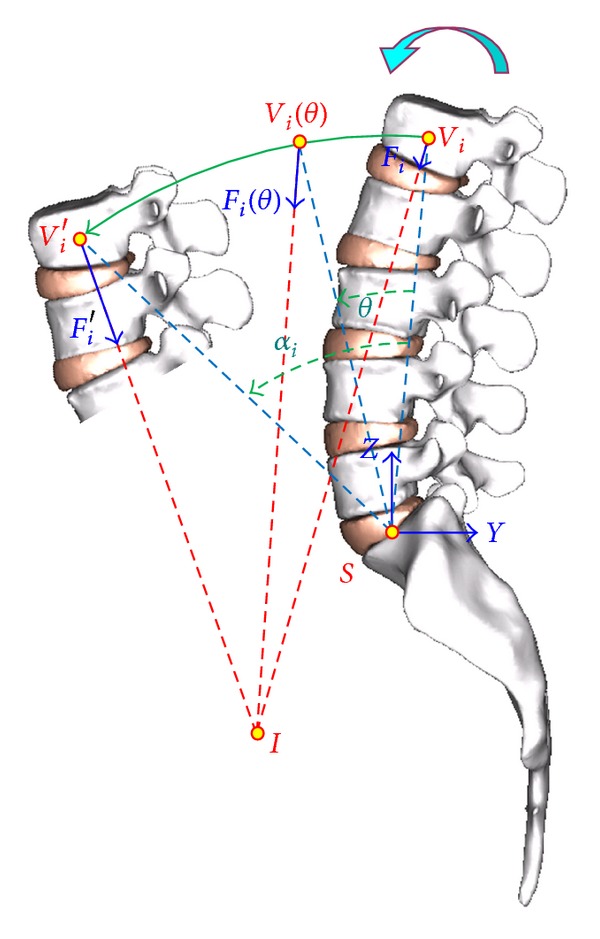
Muscle force path in flexion.

**Figure 7:**
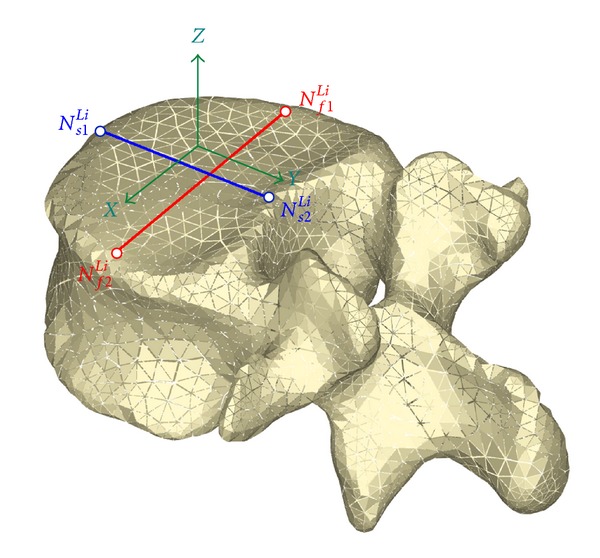
Reference points and lines for processing vertebrae mobility.

**Figure 8:**
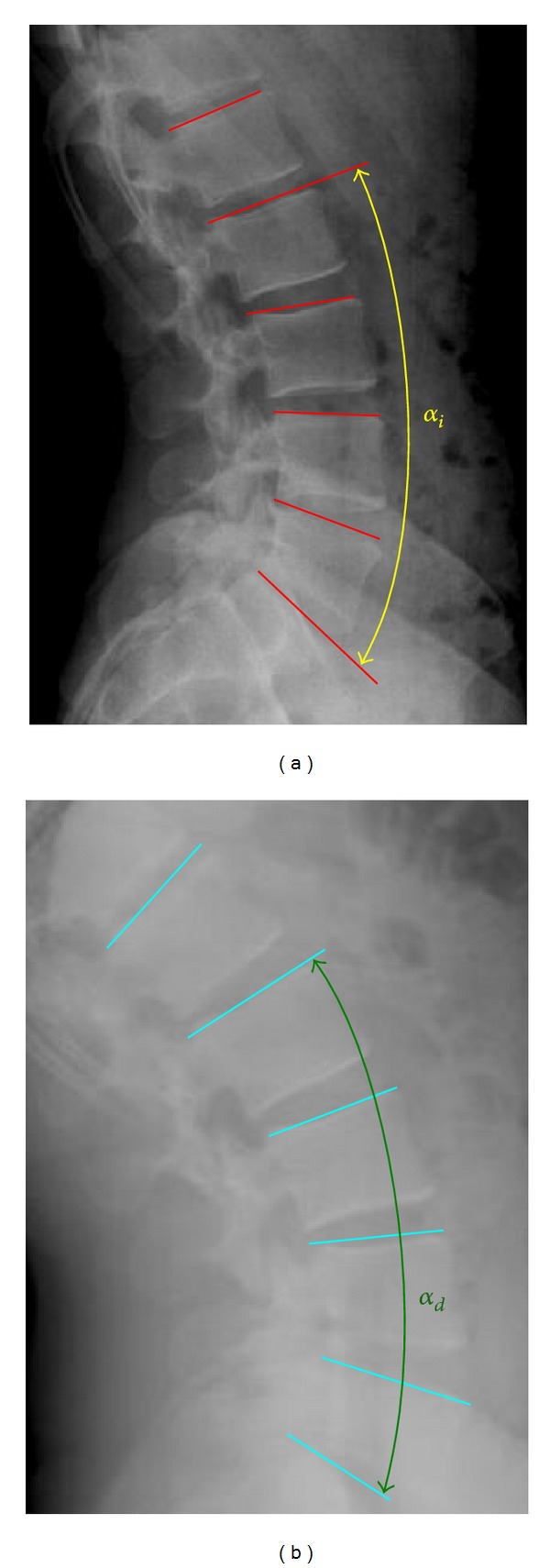
Measurements on radiological images (standing and extension).

The role of the fibres on the annulus fibrosus is essential in the biomechanical behaviour of the lumbar spine [60], its adequate modelling being fundamental. In the developed model, fibres have been added in great detail, respecting the distribution in layers, as well as the variable inclination from the interior to the exterior of the annulus (Figure 2), making it possible to obtain precise stresses distributions (Figures 11 and 12). This is very significant with regard to previous models [61–65], which excessively simplify the behaviour of the fibres.

Another important topic in the model is the anatomical accuracy of ligaments when comparing with previous works which simplify them to linear or nonlinear one-dimensional springs or truss type elements [30–34, 36] and then cannot obtain precise stress distributions or detect transverse displacements which can produce local instability. 

Moreover, the model is capable of providing reliable results of stresses values in any of the elements which form the model. This differs to the existing models which are in general limited in this aspect, when the behaviour of some elements is simplified [30–34, 36]. This is essential at the time of analysing different pathologies and when making it possible to simulate the biomechanical repercussion of the fixations, since the clinical studies [66–68] suggest that the stress concentrations in the adjacent spaces can give rise to, in the medium and long term, new pathologies in these levels. Finally, the developed model makes it possible to obtain results in a wide range of each movement, reaching the usual anatomical maximum values.

The mobility of the lumbar spine has been studied, both in vivo and in vitro using different methods [8–25]. In the radiological measurements, it is difficult to get fixed references, due to the different degrees of rotation in each X-ray, focal distance, and position of the hip and pelvis. The same is applicable to the studies with video fluoroscope or computer monitor. In addition, the mobility measurements on the same individual can vary throughout the day [24]. The studies with CT are carried out in decubitus position and in wide range of movements, above all in rotation [17]; in the studies with MRI, there are limitations in the number of cases and in the range of mobility that this technique currently allows (a maximum flexion angle of 45°) [21–23]. The studies with corpse spines [8–11] are of little value, due to the loss of flexibility and range of movements. All of this leads to a great variability in the ranges of mobility in the different published works; in addition to the fact that mobility and biomechanics vary with age [18, 25, 69] and with the underlying pathology [21, 41].

The aim of the simulation models is to fulfill the requirements of reproducibility and versatility, with the advantage of being able to repeat the study as it is a noninvasive investigation, and the initial conditions are changeable. Some authors model one or two functional units [30, 34–39, 61–63], while others model the complete lumbar spine [31–33, 46, 54, 65]. An important geometrical simplification is present in most of the models, concerning mainly to ligaments (uniaxial models with spring or truss type elements) and annulus fibrosus (number and disposition of fiber layers) [30–34, 36]. Models in which the behaviour of the intervertebral disc is simulated in a more complex way [34, 35] only consider one or two functional units instead of the complete lumbar zone. This provides results at a local level, but they cannot be extrapolated to the level of global behaviour. Some works that are dedicated to specific problems exist [39, 40] but have not managed to integrate a complete model of the lumbar spine with nonlinear behaviour.

**Figure 9:**
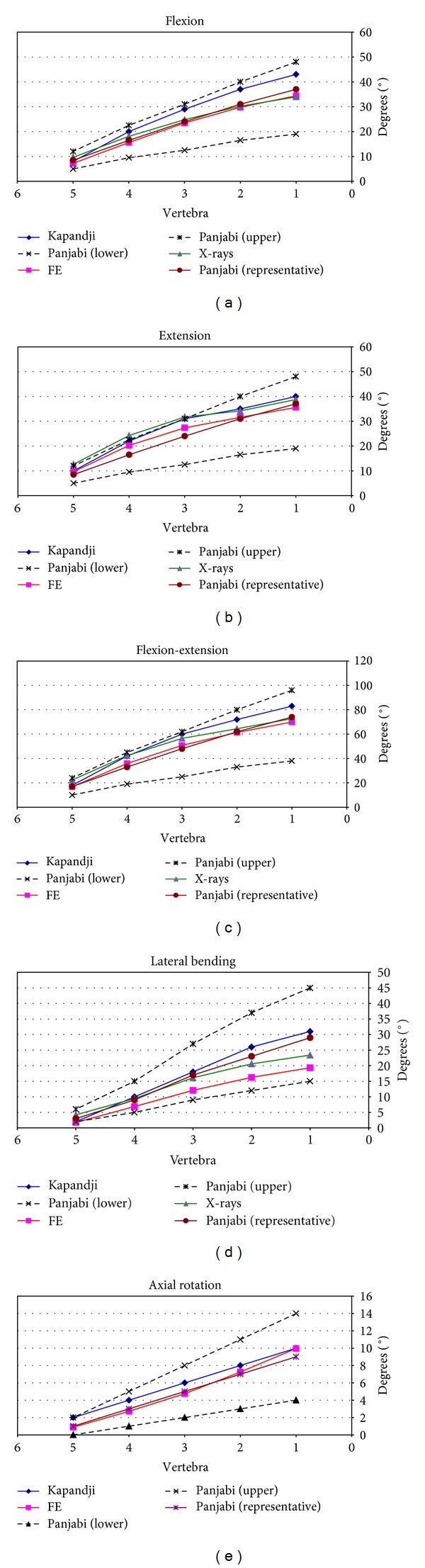
Comparison of angles in flexion, extension, flexion-extension, lateral bending, and rotation.

In the developed model, in flexion and extension a progressive movement of the vertebrae is produced as the distance to the sacrum increases, and the accumulated movements are increasing in the order L5 → L4 → L3 → L2 → L1. However, the segmental movement between two vertebrae is less in the segments nearest the sacrum (lower levels) (Figure 9(c)) according to other authors [32, 65]. The ranges obtained correspond with the average values of the radiological measurements carried out and with those of classic works [50, 52], in addition to up-to-date references [19, 70]. However, opposite results are also referenced, both in classic studies [11, 13] and in more recent ones with electrogoniometer, spectrometry, and MRI [23]. This difference could be due to the fact that in these studies flexion is limited to 45°, because of the limitations of MRI devices. Besides, the sample is not numerous enough to establish general patterns.

In lateral bending, in relative terms greater mobility is observed in the intermediate levels and in the range of maximum values accepted in the more classical references [50, 52]. Mobility is less in the upper segments coinciding with dual fluoroscope studies and MRI [23]. A greater degree of accumulated movement is observed the higher the vertebrae are (Figure 9(d)), in concordance with [23]. 

**Figure 10:**
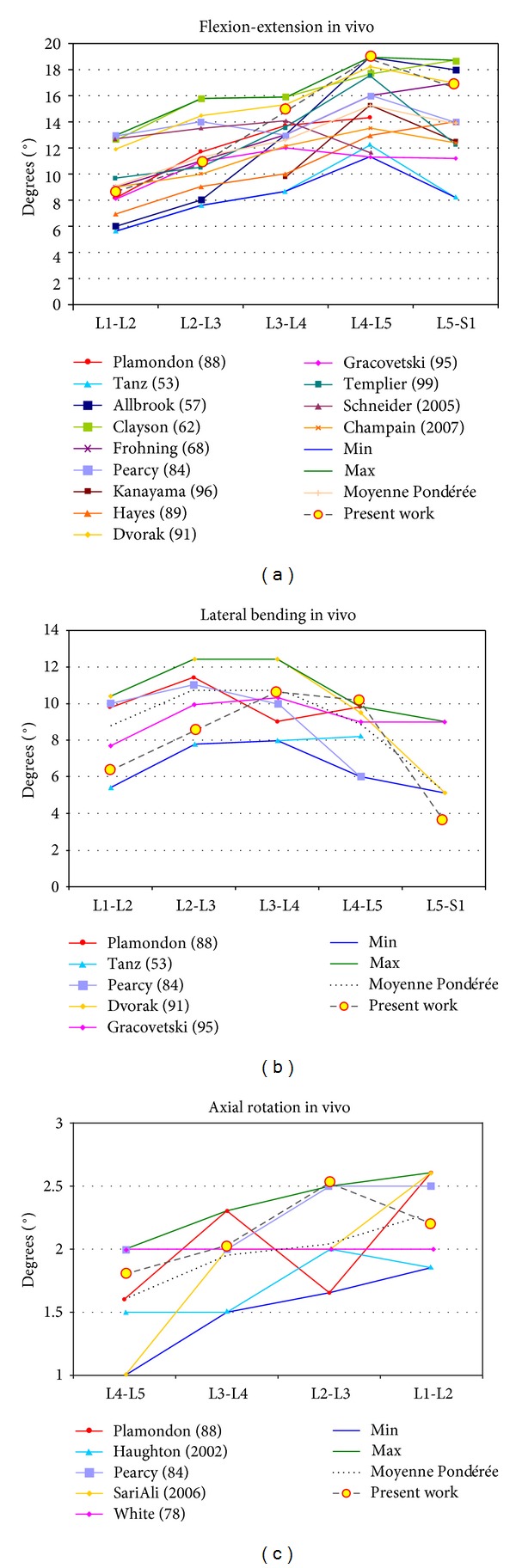
Comparison of angles in flexion-extension, lateral bending, and rotation with data from [57].

**Figure 11:**
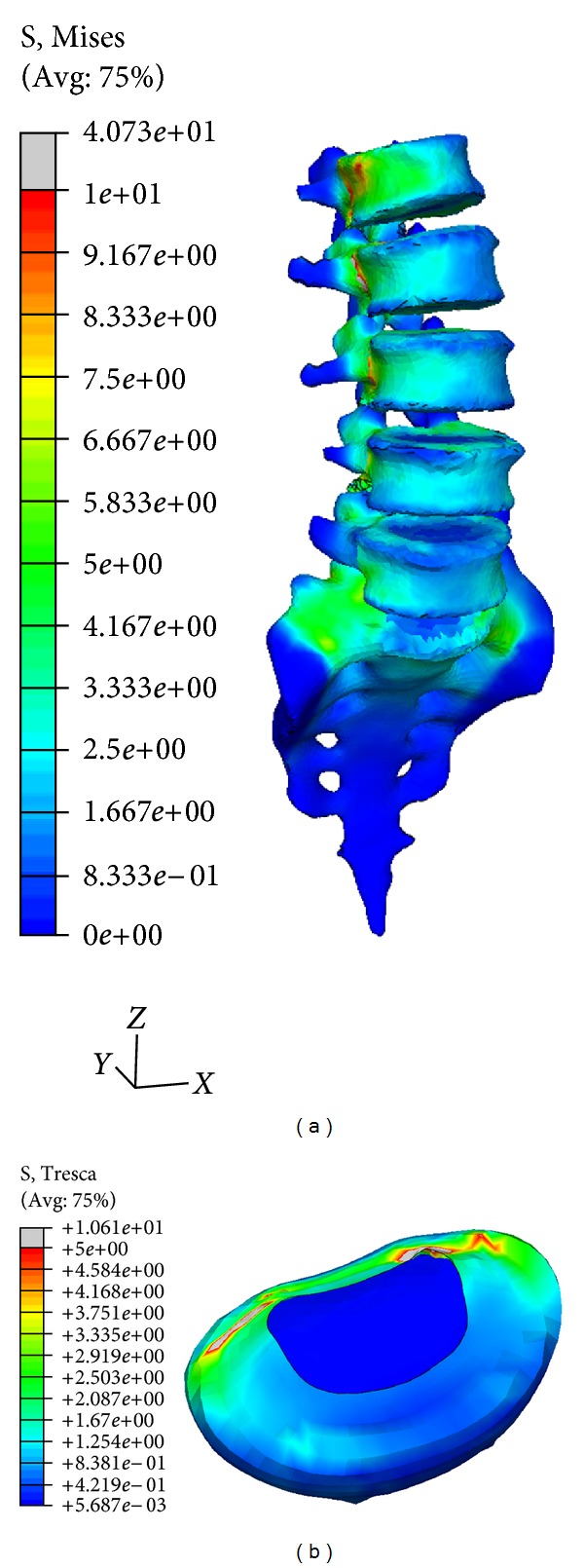
Stress distribution: (a) von Mises stress in vertebrae and sacrum (axial rotation) (MPa); (b) maximum shear stress in L5-S1disc (extension) (MPa).

**Figure 12:**
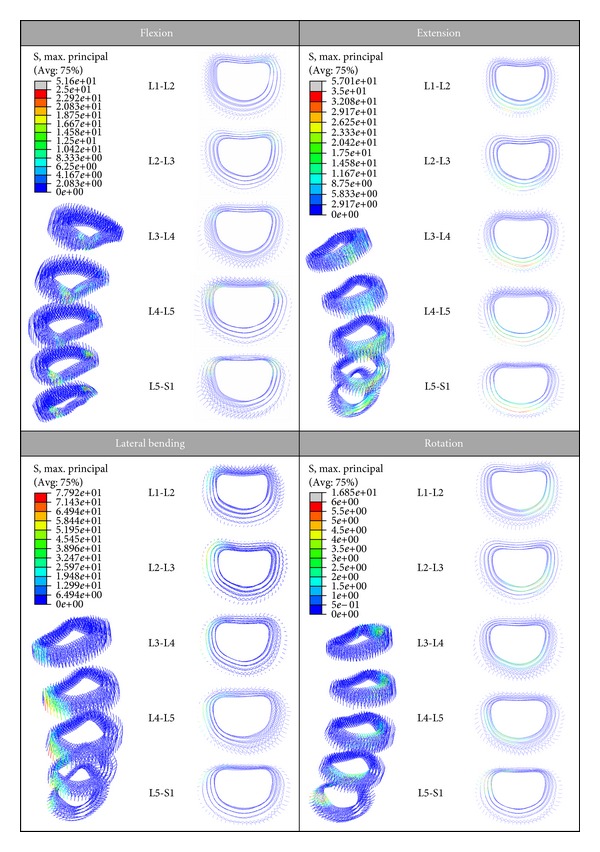
Stress distribution on the fibers of the annulus fibrosus (MPa).

**Figure 13:**
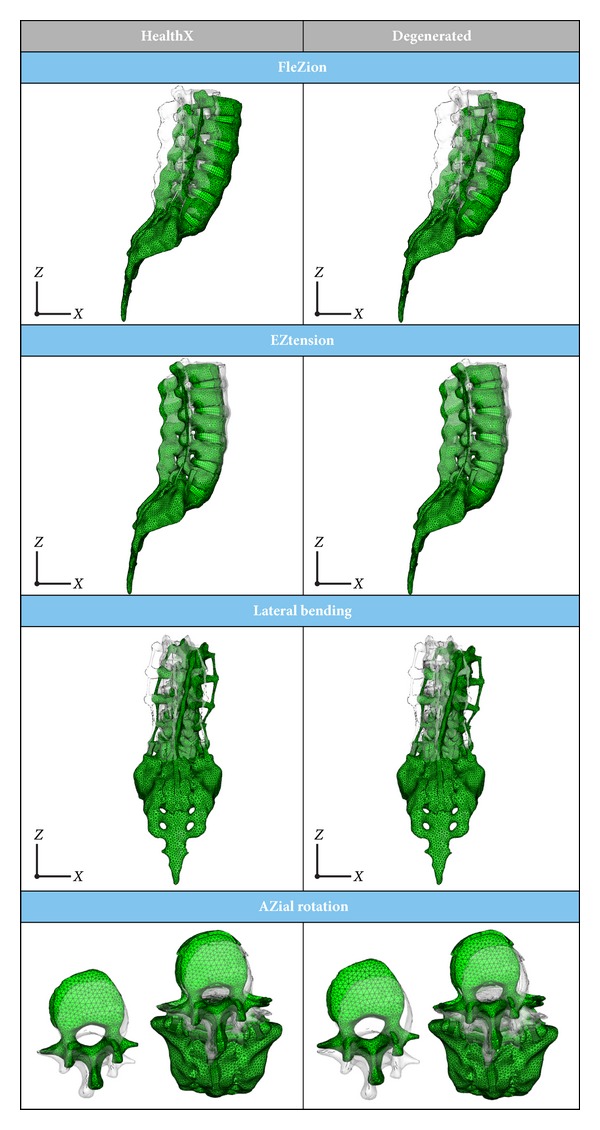
Deformed configuration: healthy model versus model with disc degeneration at L5-S1 level.

**Figure 14:**
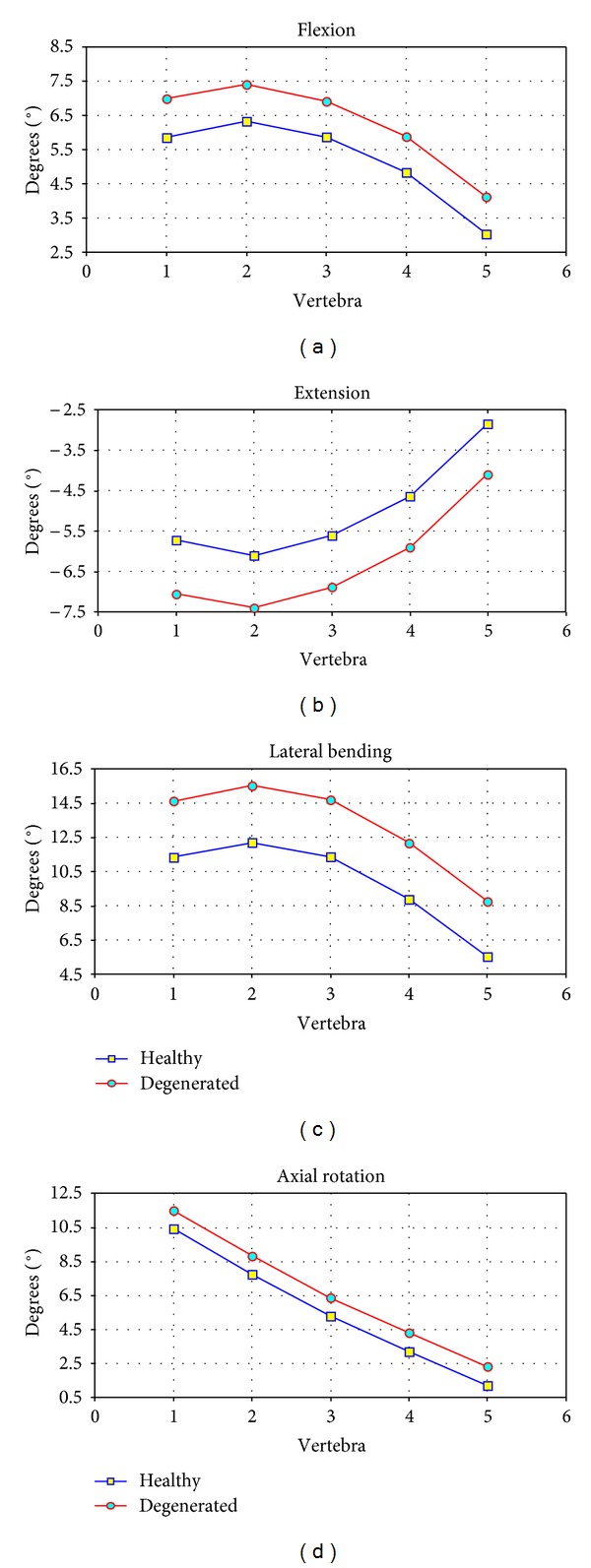
Mobility results. Comparison between healthy model and model with disc degeneration at L5-S1 level.

**Figure 15:**
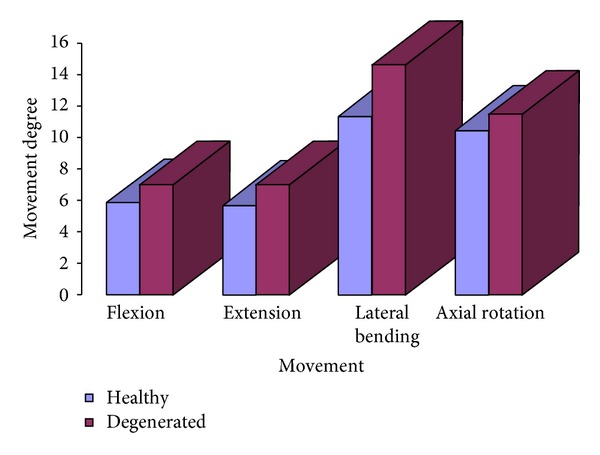
Global mobility. Difference of mobility between healthy model and model with disc degeneration at L5-S1 level.

In rotation, the comparison of twisting is more complex, as the study of this movement “in vivo” is much more difficult due to the difficulty of finding reference points. In the developed model the movements in torsion can be studied in a similar way to the rest of movements (Figure 9(e)). There is not a noticeable difference in the degrees of rotation of the different vertebrae in agreement with [23], and once again the maximum ranges are accepted in the classic references [50, 52]. Torsion has been studied in different situations and with different techniques: MRI [21–23], X-rays [12–15], three-dimensional television system [16], and CT [19]. In the majority of the studies the upper levels have a greater degree of mobility when assessing the rotation in supine position. According to [23], the different results in the torsion could be due to the different load conditions and to the position of the lumbar spine. This makes quantitative comparison difficult. Due to that dispersion of results in the different measurements, a former comparative analysis was carried out with classical references [50, 52] recognized by the majority of authors. Notwithstanding the aforementioned, in [57] a complete review of different in vivo works along more than fifty years is done, and it can be confirmed that the present results are close to the average values included in the review (Figure 10). In pathologic conditions, the obtained results agree with [56], showing an increase in the mobility at every vertebral level (Figure 15).

## 4. Conclusions

A complete three-dimensional FE model of the lumbar spine has been developed and verified, in to which all of the structures of the spine have been incorporated. This can be modified to reproduce the biomechanics in physiological and pathological conditions. Therefore making it possible to simulate the pathological conditions of hypermobility and lumbar segmental instability produced by disc degeneration, which is associated with pain of discogenic origin [14, 71, 72].

The developed model provides a valid tool for predicting the biomechanical behaviour of the lumbar spine in different conditions and is capable of reproducing a wide range of physiological movements. The model represents the first step for the analysis of the behavioural changes induced by different pathologies, allowing parametric studies for different ranges of disc degeneration.

## Figures and Tables

**Table 1:** Mechanical properties of materials.

Material	Young modulus (MPa)	Poisson coefficient	Element type	Number of elements
Outer vertebral endplates	12000	0.3	Tetrahedron	3578
Intermediate vertebral endplates	6000	0.3	Tetrahedron	2244
Centre of the vertebral endplates	2000	0.3	Tetrahedron	831
Walls of the vertebral body	12000	0.3	Tetrahedron	37205
Cancellous bone (inside vertebrae)	100	0.2	Tetrahedron	44954
Posterior vertebra	3000	0.3	Tetrahedron	47134
Cartilage	50	0.4	Wedge	3086
Annulus fibrosus	4.2	0.45	Hexahedron	8288
Nucleus pulposus*	Incompressible material		Tetrahedron	14410
Annulus fiber layers 1	360	0.3	Truss**	592
Annulus fiber layers 2	408	0.3	Truss**	592
Annulus fiber layers 3	455	0.3	Truss**	592
Annulus fiber layers 4	503	0.3	Truss**	592
Annulus fiber layers 5	550	0.3	Truss**	296

Ligament	Young modulus (MPa)	Transition strain (%)	Element type	Number of elements

Anterior longitudinal ligament	7.8	12.0	Wedge**	9046
	20.0			
Posterior longitudinal ligament	10.0	11.0	Wedge**	3844
	50.0			
Ligamentum flavum	15.0	6.2	Tetrahedron**	3042
	19.0			
Intertransverse ligament	10.0	18.0	Tetrahedron**	6678
	59.0			
Capsular ligament	7.5	25.0	Membrane**	3220
	33.0			
Interspinous ligament	8.0	20.0	Tetrahedron**	2856
	15.0			
Supraspinous ligament	10.0	14.0	Tetrahedron**	2657
	12.0			
Iliolumbar ligament	7.8	12.0	Wedge**	816
	20.0			

*C_01_ = 0.0343 MPa; *C*_10_ = 0.1369 MPa. An elastic analysis with Young modulus of 1.0 MPa and Poisson ratio of 0.49 was carried out with similar results and a volume change less than 0.6%.

**Only tension.

**Table 2:** Mechanical properties of degenerated disc.

Material	Young modulus (MPa)	Poisson coefficient	Element type	Number of elements
Annulus fibrosus	6.0	0.35	Hexahedron	8288
Nucleus pulposus*	1.3	0.4	Tetrahedron	14410
Annulus fiber layers 1	36.0	0.3	Truss**	592
Annulus fiber layers 2	40.8	0.3	Truss**	592
Annulus fiber layers 3	45.5	0.3	Truss**	592
Annulus fiber layers 4	50.3	0.3	Truss**	592
Annulus fiber layers 5	55.0	0.3	Truss**	296

*Elastic material (compressible).

**Only tension.

**Table 3:** Results from the radiological measurements.

Vertebra	Flexion (°)		Extension (°)		Lateral bending (°)	
	Mean	Standard deviation	Mean	Standard deviation	Mean	Standard deviation
L1	33.94	4.91	38.73	4.29	23.40	2.39
L2	30.25	3.93	34.17	4.29	20.08	2.55
L3	24.78	6.20	31.70	4.28	16.12	1.38
L4	18.09	6.83	24.25	5.24	9.45	1.33
L5	9.69	4.50	12.66	4.06	4.21	0.63

## Abbreviations

FE: Finite elements
3D: Three-dimensional
TV: Television
CT: Computed tomography
NMR: Nuclear magnetic resonance
L1: First lumbar vertebrae
L2: Second lumbar vertebrae
L3: Third lumbar vertebrae
L4: Fourth lumbar vertebrae
L5: Fifth lumbar vertebrae
S1: First sacral vertebrae
FEM: Finite-element method
MRI: Magnetic resonance imaging.
